# Supplementary material for: Identification of photoperiod-induced specific miRNAs in the adrenal glands of Sunite sheep (Ovis aries)
Source: Front Vet Sci. 2022 Jul 22;9:888207. doi: 10.3389/fvets.2022.888207 (PMC9354845; doi:10.3389/fvets.2022.888207)
Supplement: Supplementary file 1 [file Data_Sheet_1.ZIP › Supplementary file/miRDeep2 file.zip!/miRDeep2 file/Know_Structure/SPLP42A2/oar-mir-30a.pdf]

miRBase precursor : oar-mir-30a
Total read count : 97320
oar-miR-30a-5p read count : 89761
oar-miR-30a-3p read count : 7558
remaining reads : 1

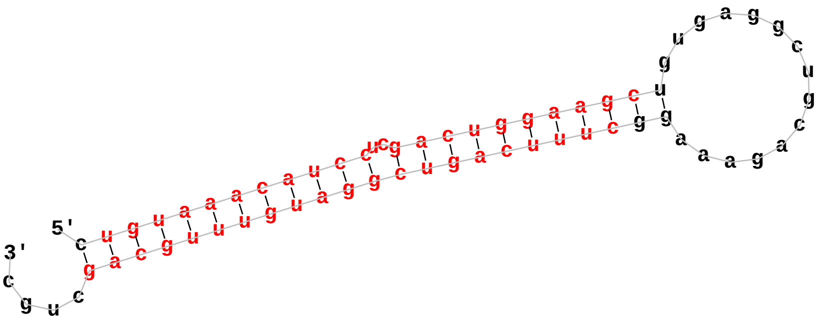


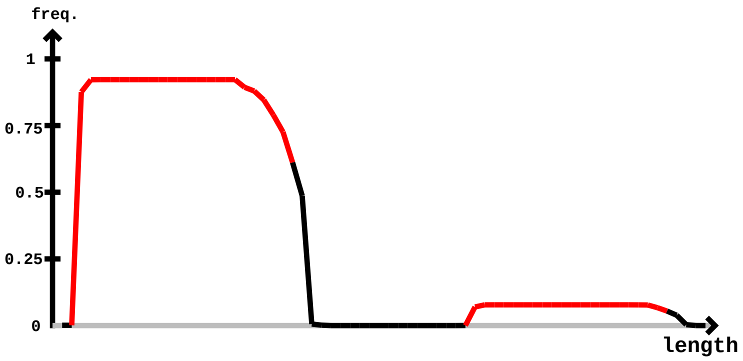


oar-miR-30a-3p

oar-miR-30a-5p

| 5'- cuguaaacauccucgacuggaagcugugaggcugcagaaaggcuuucagucggauguuugcagcugc -3' | exp | | |
|---|---|---|---|
| ((((((((((((..(((((((((((..............)))))))))))))))))))))))....  | reads | mm | sample |
| cuguaaacauccuUgacu................................................ | 1 | 1 | seq |
| cuguaaacauccuUgacug............................................... | 1 | 1 | seq |
| cuguaaacauccuUgacugg.............................................. | 1 | 1 | seq |
| cuguaaacauccucgacugg.............................................. | 6 | 0 | seq |
| cuguaaacauccuUgacugga............................................. | 2 | 1 | seq |
| cuguaaacauccucgacugga............................................. | 19 | 0 | seq |
| cuguaaacauccuUgacuggaa............................................ | 1 | 1 | seq |
| cuguaaacauccucgacuggaa............................................ | 14 | 0 | seq |
| cuguaaacauccuUgacuggaag........................................... | 2 | 1 | seq |
| cuguaaacauccucgacuggaag........................................... | 11 | 0 | seq |
| cuguaaacauccuUgacuggaagc.......................................... | 3 | 1 | seq |
| cuguaaacauccucgacuggaagc.......................................... | 20 | 0 | seq |
| cuguaaacauccuUgacuggaagcu......................................... | 15 | 1 | seq |
| cuguaaacauccucgacuggaagcu......................................... | 9 | 0 | seq |
| .uguaaacauccuUgacu................................................ | 2519 | 1 | seq |
| .uguaaacauccucgacu................................................ | 297 | 0 | seq |
| .uguaaacauccuUgacug............................................... | 1040 | 1 | seq |
| .uguaaacauccucgacug............................................... | 313 | 0 | seq |
| .uguaaacauccucgacugg.............................................. | 1388 | 0 | seq |
| .uguaaacauccuUgacugg.............................................. | 1839 | 1 | seq |
| .uguaaacauccuUgacugga............................................. | 1078 | 1 | seq |
| .uguaaacauccucgacugga............................................. | 4404 | 0 | seq |
| .uguaaacauccuUgacuggaa............................................ | 1123 | 1 | seq |
| .uguaaacauccucgacuggaa............................................ | 5010 | 0 | seq |
| .uguaaacauccucgacuggaag........................................... | 7210 | 0 | seq |
| .uguaaacauccuUgacuggaag........................................... | 3884 | 1 | seq |
| .uguaaacauccucgacuggaagc.......................................... | 9895 | 0 | seq |
| .uguaaacauccuUgacuggaagc.......................................... | 2113 | 1 | seq |
| .uguaaacauccucgacuggaagcu......................................... | 16511 | 0 | seq |
| .uguaaacauccuUgacuggaagcu......................................... | 26361 | 1 | seq |
| .uguaaacauccucgacuggaagcug....................................... | 27 | 0 | seq |
| .uguaaacauccuUgacuggaagcug....................................... | 93 | 1 | seq |
| .uguaaacauccuUgacuggaagcugu...................................... | 22 | 1 | seq |
| .uguaaacauccucgacuggaagcugu...................................... | 3 | 0 | seq |

```
                                         oar-miR-30a-3p
 oar-miR-30a-5p
cuguaaacauccucgacuggaagcugugaggcugcagaaaggcuuucagucggauguuugcagcugc

.uguaaacauccucgacuggaagcugugaggc..................................    1      0    seq
..guaaacauccuUgacug................................................   2      1    seq
..guaaacauccuUgacugg...............................................   4      1    seq
..guaaacauccucgacugg...............................................   2      0    seq
..guaaacauccuUgacugga..............................................   7      1    seq
..guaaacauccucgacugga..............................................   3      0    seq
..guaaacauccucgacuggaa.............................................   10     0    seq
..guaaacauccuUgacuggaa.............................................   11     1    seq
..guaaacauccuUgacuggaag............................................   50     1    seq
..guaaacauccucgacuggaag............................................   9      0    seq
..guaaacauccuUgacuggaagc...........................................   59     1    seq
..guaaacauccucgacuggaagc...........................................   24     0    seq
..guaaacauccucgacuggaagcu..........................................   175    0    seq
..guaaacauccuUgacuggaagcu..........................................   3734   1    seq
..guaaacauccuUgacuggaagcug.........................................   263    1    seq
..guaaacauccucgacuggaagcugu........................................   1      0    seq
..guaaacauccuUgacuggaagcugu........................................   115    1    seq
...uaaacauccuUgacuggaagc...........................................   1      1    seq
...uaaacauccucgacuggaagcu..........................................   9      0    seq
...uaaacauccuUgacuggaagcu..........................................   19     1    seq
...uaaacauccuUgacuggaagcug.........................................   16     1    seq
...uaaacauccucgacuggaagcug.........................................   1      0    seq
...uaaacauccucgacuggaagcugu........................................   1      0    seq
...uaaacauccuUgacuggaagcugu........................................   7      1    seq
....aaacauccucgacuggaagcu..........................................   1      0    seq
....aaacauccuUgacuggaagcug.........................................   1      1    seq
....aaacauccuUgacuggaagcugu........................................   1      1    seq
.........................................gcuuucagucggauguuuAc......   4      1    seq
.........................................gcuuucagucggauguuuAca.....   13     1    seq
.........................................gcuuucagucggauguuuAcag....   8      1    seq
.........................................gcuuucagucggauguuuAcagc...   4      1    seq
..........................................Auuucagucggauguuu........   1      1    seq
..........................................cuuucagucggauguuu........   19     0    seq
..........................................cuuucagucggauguuuA.......   21     1    seq
..........................................cuuucagucggauguuug.......   13     0    seq
..........................................cuuucagucggauguuuAc......   749    1    seq
..........................................cuuucagucggauguuugc......   159    0    seq
..........................................cuuucagucggauguuuAca.....   1084   1    seq
..........................................cuuucagucggauguuugca.....   110    0    seq
..........................................cuuucagucggauguuugcag....   340    0    seq
..........................................cuuucagucggauguuuAcag....   1089   1    seq
..........................................cuuucagucggauguuuAcagc...   2228   1    seq
..........................................cuuucagucggauguuugcagc...   884    0    seq
..........................................cuuucagucggauguuugcagcu..   69     0    seq
...........................................uuucagucggauguuug.......   1      0    seq
...........................................uuucagucggauguuugc......   20     0    seq
...........................................uuucagucggauguuuAc......   53     1    seq
...........................................uuucagucggauguuuAca.....   28     1    seq
...........................................uuucagucggauguuugca.....   3      0    seq
...........................................uuucagucggauguuugcag....   30     0    seq
...........................................uuucagucggauguuuAcag....   38     1    seq
...........................................uuucagucggauguuugcagc...   148    0    seq
...........................................uuucagucggauguuuAcagc...   181    1    seq
...........................................uuucagucggauguuugcagcu..   250    0    seq
...........................................uuucagucggauguuugcagcug.   8      0    seq
............................................uucagucggauguuugcagc...   3      0    seq
```
